# Imaging infection amid inflammation

**DOI:** 10.1172/JCI162885

**Published:** 2022-09-15

**Authors:** Patrick C. Seed

A common clinical conundrum is localizing sites of deep infection and differentiating sterile from nonsterile inflammation. Distinguishing infection and other forms of inflammation is highly consequential; this affects subsequent diagnostic testing, interventional procedures, prognoses, and the medical therapies employed, including the use of antiinfectives.

PET imaging already aids in localizing sites of concern for infection and differentiating them from sterile inflammation based on the biochemical and metabolic activity of the sites of interest. The commonly used PET radiotracer 2-deoxy-2-[18F]fluoro-D-glucose (FDG) is taken up in tissue sites with increased metabolic activity, suggestive of sites of inflammation that may include infection (1). In particular, microbes and white blood cells typically have higher glucose utilization than surrounding tissues. However, human and microbial cells uptake the labeled glucose for metabolism, and the tracer with PET cannot always definitively differentiate between areas with and without infection. Other metabolically active sites without infection or inflammation, as found in cancer, organ injury, or inherently metabolically demanding organs, such as the liver and heart, also readily uptake the tracers such as FDG. PET tracers exclusively taken up by microbes would facilitate differentiation of infection and sterile inflammation, aiding in clinical decision-making.

In this issue of the *JCI*, Lee, Jacome, and colleagues extended their investigation of an antibiotic-based radiotracer, [^13^C]-trimethoprim, combined with PET to localize the site of potential infection and to distinguish sterile and nonsterile inflammation (2). Trimethoprim, a clinically used antibiotic, selectively binds to bacterial dihydrofolate reductase (DHFR) to block folate metabolism and impair nucleotide synthesis, thus arresting bacterial growth. When labeled for PET imaging, the accumulated trimethoprim serves as a beacon from the site of an infection.

The authors performed a pilot study involving human participants in which [^11^C]-trimethoprim PET imaging localized to sites of presumed and biopsy-proven infection, which was also confirmed by other imaging modalities, including CT and MRI. The authors further demonstrated that, compared with FDG, the trimethoprim-based radiotracer did not visually enhance sites of primary and metastatic cancer.

Other bacteria-specific radiotracers, including antibiotics and specific metabolites, have been proposed for PET imaging to localize infections (3, 4). However, a major limitation of using antibiotic-based tracers lies in resistance to the antibiotic agent. If a microbe does not uptake and retain the antibiotic tracer, it is rendered useless. Does trimethoprim resistance limit using the ^11^C-trimethoprim tracer to detect bacterial infections? Lee, Jacome, and colleagues demonstrated that, despite trimethoprim resistance, medically relevant bacteria remained labeled with the antibiotic-based tracer (2). The investigators used whole-genome sequencing of clinically relevant bacterial isolates to show that the DHFR gene commonly exists in multiple copies, including variants that confer drug resistance alongside drug-sensitive variants. Although bacteria may have been phenotypically resistant to the trimethoprim, they continued to express the drug-sensitive enzyme and remained capable of binding the PET radiotracer.

While trimethoprim-based PET radiotracers open opportunities to detect deep invasive bacterial infections, barriers still exist that limit the more generalized use of these and other antibiotic-based radiotracers. Antibiotics have multiple specificities for different types and species of organisms that may influence the sensitivity and specificity of imaging different types of infections. Conversely, many antibiotics are nonspecific, taken up by multiple species of bacteria; this limits the ability of radiotracers to provide specific information about the exact etiology of an infection localized by PET or its antibiotic susceptibility profile. Thus, a positive PET scan using an antibiotic-based tracer may still require invasive sampling of the presumed infection to obtain prognostically and therapeutically important information. Despite these limitations, antibiotic-based PET radiotracers, such as those using trimethoprim, may provide an important tool for clinicians to noninvasively narrow the list of diagnoses and necessary diagnostic and therapeutic next steps.

## Version 1 09/15/2022

Electronic publication
